# A Case of Macrophage Activation Syndrome Manifesting as the Initial Presentation of Systemic Lupus Erythematosus

**DOI:** 10.7759/cureus.26375

**Published:** 2022-06-27

**Authors:** Amar Suwal, Biraj Shrestha, Anish Paudel, Rubina Paudel, Sijan Basnet

**Affiliations:** 1 Internal medicine, Reading Hospital-Tower Health, Reading, USA; 2 Internal Medicine, Reading Hospital-Tower Health, Reading, USA

**Keywords:** major hyperferritinemia, cytokine release storm, corticosteroid, systemic lupus erythematosis, macrophage activation syndrome (mas), hemophagocytic lymphohistiocytosis (hlh)

## Abstract

Macrophage activation syndrome (MAS) is a potentially fatal complication of an autoimmune rheumatologic disease characterized by overwhelming inflammation, multiorgan failure, and high mortality if untreated. We report a rare case of a 56-year-old man who presented with fever for three weeks and had a constellation of clinical features and laboratory findings, meeting the diagnostic criteria for systemic lupus erythematosus (SLE) and SLE-associated MAS. He was treated with high dose intravenous corticosteroid and hydroxychloroquine, resulting in resolution of fever and dramatic clinical improvement.

## Introduction

Macrophage activation syndrome (MAS) is a life-threatening complication of an autoimmune rheumatologic disease that falls in the spectrum of dysregulated immune disorders called hemophagocytic lymphohistiocytosis (HLH ) [1]. MAS/HLH is characterized by overwhelming inflammation and cytokine storm resulting from unchecked activation/proliferation of macrophages and T lymphocytes, ultimately resulting in multiorgan failure if untreated [1-3]. While MAS has been reported in almost any rheumatologic condition, the reported incidence of MAS in SLE is about 0.9% to 4.6% [4], with mortality reaching up to 35% [5-7]. However, this incidence of MAS in SLE could be underestimated due to being misdiagnosed as a flare-up or complication of SLE [1]. We report a case of MAS presenting as an initial manifestation of a newly diagnosed SLE

## Case presentation

**Figure 1:**
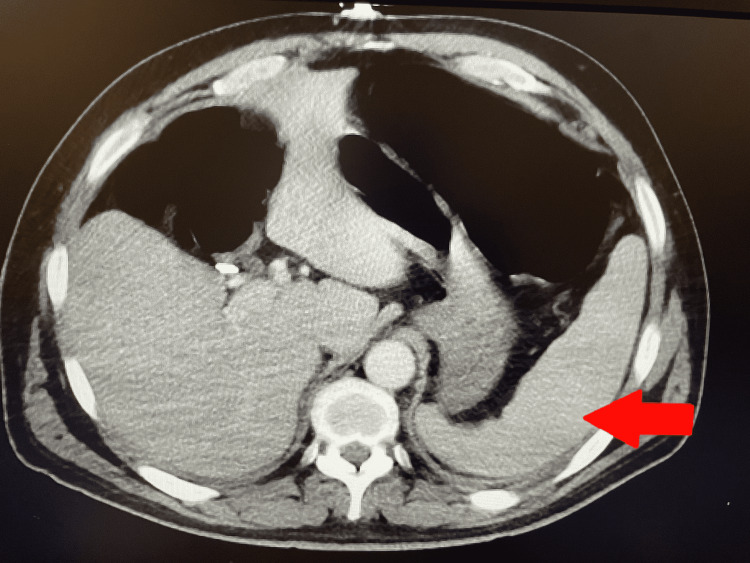
CT Abdomen with contrast showing splenomegaly.

A 56-year-old man with a past medical history of hypertension and benign prostate hyperplasia presented to the emergency department with a 3-week history of fever, night sweats, fatigue, loss of appetite, and 15 lbs. weight loss. He denied any personal or family history of rheumatic diseases in the past. His review of systems was negative for any photosensitivity, rash, alopecia, joint swelling, and oral ulcers. His home medications included Amlodipine 10 mg daily, Hydrochlorothiazide 12.5 mg daily, and Tamsulosin 0.4 mg daily. His vitals were within normal limits, with no fever on admission. His physical examination was unremarkable. Pertinent lab findings were leukopenia (white blood cell (WBC)) count of 3000/uL with neutrophil (70%), lymphocyte (21%), eosinophil (1.6%), and basophil (0.6%), mild normocytic anemia (hemoglobin:12.6 g/dl), elevated erythrocyte sedimentation rate (ESR), and C-reactive protein (CRP), and mildly elevated ferritin (503 ng/ml) and lactate dehydrogenase (LDH of 260 IU/L). His total bilirubin was within normal limits (0.6 mg/dl). Computed tomography (CT) of the chest showed mediastinal and upper abdominal lymphadenopathy, and subsequent CT abdomen to look for the extent of lymphadenopathy showed moderate splenomegaly (16.5 cm) (Figure 1). For leukopenia with generalized lymphadenopathy, flow cytometry was ordered. However, before completing his clinical investigation, he insisted on going home as he reported feeling better and wanted to follow up with the hematology clinic as an outpatient. His symptoms were attributed to a viral syndrome, and he was discharged after his initial workup for infectious etiology (Table 1) was negative.

**Table 1:** shows extensive infectious disease workup, which was unremarkable. IgG Ab was detected for parvovirus B19, EBV, and HSV, indicating infection in the past. RSV: respiratory syncytial virus HIV: human immunodeficiency virus TB: tuberculosis EBV: Epstein Barr virus HSV: herpes simplex virus Hep: hepatitis Ab: Antibody

Test	Result	Reference Range
COVID test	Negative	Negative
RSV, Influenza	Negative	Negative
Blood culture	No growth	No growth
HIV Rapid Antibody 1 / 2	Non-reactive	Non-reactive
HIV Rapid p24 antigen	Non-reactive	Non-reactive
Urinary Antigen Strep Pneumoniae	Negative	Negative
Legionella Urinary antigen	Negative	Negative
Ehrlichia and Anaplasma Ab	<1:80	<1:80
Mononucleosis Ab	Negative	Negative
Parvovirus B12 IgG	6.3	<0.9
Cytomegalovirus PCR	Not detected	Not detected
Blood Parasite Smear( Babesia)	No Parasite found	Not detected
Quantiferon TB	Negative	Negative
Lyme Ab screen, Ig G, and IgM	Negative	Negative
Epstein Bar VCA- IgM	<36	<36 IU/ml
EBV VCA IgG	>750	<18 IU/ml
EBV Nuclear Ab (IgG)	228	<18 IU/ml
Lymph node- fungal culture	Negative	Negative
Lymph node- AFB	Negative	Negative
HSV type 1 / 2 Ab combined IgG	>22.4	<0.9 IV
Hep B Surface Ag, Hep B Core IgM Ab, Hep A IgM, Hep C Ab	All Non-reactive	Non-reactive
Sputum culture and gram stain	Negative	Negative

Two days following discharge, the patient presented after a syncopal event while trying to get up. Since his recent discharge, he reported ongoing febrile episodes, extreme weakness, and poor oral intake at home. His examination showed a fever of 103°F and bilateral inguinal lymphadenopathy; otherwise, no rash or joint involvement. His blood work still showed pancytopenia (Table 2). Hemolysis workup showed a positive Coombs test but no signs of active hemolysis with a normal haptoglobin of 160 mg/dl, total bilirubin of 0.7 mg/dl, and direct bilirubin of 0.1 mg/dl. A peripheral blood smear showed pancytopenia and ruled out any hemolysis. His infectious workup continued to be negative, including a repeat coronavirus disease 2019 (COVID) test and repeat blood culture. He was empirically placed on intravenous vancomycin and cefepime. Later, after a negative blood culture, the antibiotic was narrowed to cefepime for neutropenic fever (absolute neutrophil count less than 500/ul), while his fever continued. Due to persistent fever, he further underwent workup for other possible causes of fever of unknown origin, including malignancy, lymphoma, and autoimmune rheumatic disease.

**Table 2:** shows immunological investigation during the index admission, along with an improved trend in ferritin, cytopenia, and inflammatory markers during the hospital course after the initiation of therapy LDH: lactate dehydrogenase ANA = antinuclear antibody, anti-dsDNA = anti-double-stranded DNA S-IL2R: soluble interleukin-2 receptor NK cell activity: natural killer cell activity ESR: erythrocyte sedimentation rate CRP: C-reactive protein C3 = complement component 3, C4 = complement component 4,

Test	Admission	Peak value (during the hospital stay)	Discharge	Reference Range
LDH	336	383	155	85-227U/L
Ferritin	1487	3968	881	26-388 ng/ml
ANA	1:640			<1:40
Anti-Ds DNA	1:160			<1:10
Anti-Smith	2			0-40 U/ml
Lupus anticoagulant	Not detected			-
S-IL2R	6772			266.5-1410.4 pg/ml
NK cell activity	depressed			-
ESR	55			0 - 20 mm/hr
WBC	1.4	15.5	7.5	4.8 - 10.8 10E3/uL
Hemoglobin	11.8	12.3	10.9	14.0 - 17.5 g/dL
Platelet	98	110	224	130 - 400 10E3/uL
Anticardiolipin Antibody IGA	9			0-11 GAL
Anticardiolipin Antibody IgG	15			0-14 GPL
Anticardiolipin Antibody Ig M	58			0-12 MPL
Β2 glycoprotein IgM	37			0-20
CRP	12.44	4.62	2.41	0.00 - 0.90 mg/dL
Immunoglobulin G	1632			768-1632 mg/dl
Immunoglobulin M	339			35-263 mg/dl
Total complement	30			31 - 60 U/mL
C3	60			82 - 185 mg/dL
C4	3.6			15.0 - 53.0 mg/dL

Autoimmune workup was confirmatory for SLE with positive ANA titer and ds-DNA along with low complements (C3 and C4) (Table 2). Flow cytometry didn't reveal increased Blasts. Bone marrow biopsy revealed normocellular marrow without any hemophagocytosis, no atypical lymphoid infiltrate, or blast increase. Additionally, he had reduced natural killer (NK) cell activity and elevated soluble interleukin-2 receptor, and was thus diagnosed with HLH and placed on intravenous methylprednisone 250mg every 12 hours for three days starting on the 10th day of this hospitalization, followed by 60mg daily. Also, the patient was started on hydroxychloroquine 200mg twice a day. Subsequently, from the next day of this high-dose steroid, his ferritin and CRP levels trended down, and his cytopenia started to improve, along with the resolution of fever (Table 2). He has eventually discharged on hydroxychloroquine 200mg twice daily and prednisone 80mg daily, with a plan for 10mg weekly taper and outpatient follow-up with the rheumatology and hematology clinic.

## Discussion

Macrophage Activation Syndrome is characterized by macrophage overactivation, resulting in hemophagocytosis, thus placing MAS under the umbrella of Hemophagocytic Lymphohistiocytosis (HLH). Scott and Robb-Smith first described HLH in 1939 [8]. It can either result from genetic mutations affecting cytotoxic function (familial HLH) or be caused by acquired triggers like malignant, infectious, or rheumatologic disorders that disrupt the immune homeostasis [2]. MAS is used to designate the acquired HLH resulting specifically from autoimmune rheumatologic diseases [2-3]. Regardless of the cause, the underlying mechanism is speculated to be an inability of cytotoxic cells (NK cells and cytotoxic T lymphocytes) to eliminate the trigger for inflammation, causing failure to contain the inflammation [3]. This results in persistent antigen stimulation and excessive activation of macrophages/T-lymphocytes, leading to a massive release of proinflammatory cytokines (cytokine storm), subsequent immune dysregulation, and multiorgan failure [3].

Our patient presented with a high-grade fever unresponsive to empiric antibiotics, pancytopenia, lymphadenopathy/splenomegaly, hyperferritinemia, reduced NK cell activity, and elevated soluble interleukin-2 receptor, and thus met 6 out of 8 criteria per HLH-2004 trial [9] and had an H score of 204 with 88-93 % probability of HLH [10]. We think his HLH was the initial presentation of his previously undiagnosed SLE as he also met the diagnostic ACR- EULAR criteria for SLE (positive ANA titer 1:640), >10 points. (fever- 2, thrombocytopenia- 4, pericardial effusion- 5, low C3 and Low C4- 4, anti-double-stranded DNA antibody (anti-dsDNA)- 6, APL antibody- 2- total 23). It is not uncommon for MAS secondary to SLE to present without clinical evidence of lupus exacerbation [11]. Hence, the patient was diagnosed with MAS secondary to SLE after ruling out other acquired causes of HLH, including infection and malignancy.

Diagnosis of MAS is based on HLH-2004 clinical criteria, which require at least the presence of molecular diagnosis consistent with HLH or five out of eight findings that include fever >38.5 °C, splenomegaly, peripheral blood cytopenias (at least any two), hypertriglyceridemia, hemophagocytosis in either bone marrow, lymph node, spleen or liver biopsy, low or absent natural killer (NK) cell activity, hyperferritinemia, and elevated soluble interleukin-2 receptor alpha chain (CD25) or elevated chemokine (C-X-C motif) ligand 9 (CXCL9) [1]. Additional common findings include transaminitis, coagulopathy, hyponatremia, hypoalbuminemia, elevated lactate dehydrogenase (LDH), C-reactive protein, and D-dimer [12-13]. MAS should always be in the differential in patients presenting with a triad of persistent fever, hepatosplenomegaly, and cytopenias, especially with elevated ferritin [14]. Many of its features (fever, hepatosplenomegaly, lymphadenopathy, cytopenia, coagulopathy, and elevated CRP) overlap with other severe illnesses like sepsis and malignancy [12-13]. Moreover, these same disorders can trigger HLH, further complicating the diagnosis. It can also be challenging to differentiate MAS from an SLE flare; however, some findings like hyperferritinemia, hemophagocytosis, hypofibrinogenemia with low ESR, hypertriglyceridemia, and decreased NK cell activity are more specific to HLH and can help distinguish MAS from SLE flare [12]. However, clinicians should be mindful that many distinctive features, like hyperferritinemia, and hemophagocytosis, often occur in the later stages of MAS [12,13]. 

Treating the inciting etiology, although necessary, is often not enough in the treatment of acquired HLH. However, MAS seems rather unique as it usually responds quite well to high-dose corticosteroids alone, just like in our patient [2]. Therefore, up to a maximum daily dose of 1 gm methylprednisone for three days followed by a maintenance dose with tapering guided by clinical improvement has been recommended [9]. Additionally, cyclosporin therapy and other anti-cytokine agents targeting IL-1 and Il-6 have been employed successfully to treat MAS [15]. However, there should be a low threshold for treatment escalation per HLH -94 protocol (i.e., etoposide and dexamethasone) in case of clinical deterioration [15]. Therefore, early recognition of MAS is crucial for the timely initiation of appropriate treatment modalities, including immunosuppressive and anti-cytokine agents [13], to improve the high mortality/ morbidity associated with MAS/HLH. 

## Conclusions

MAS is a life-threatening complication of SLE, usually occurring during the active disease course while occasionally manifesting secondary to new-onset SLE. Diagnosis of HLH/MAS should be entertained in a patient presenting with a triad of persistent fever, hepatosplenomegaly, and cytopenias after ruling out infection, especially in the setting of elevated ferritin. Checking for NK cell activity and IL2a levels helps diagnose HLH, especially in early-stage when hemophagocytosis and other distinctive features might be absent; however, if unavailable, H- score (a validated score based on routinely available tests) should be used. Early recognition of MAS is crucial for the timely initiation of appropriate treatment modalities including immunosuppressive and anti-cytokine agents, to improve the high mortality/ morbidity associated with MAS/HLH. 
